# Representation Matters: Demographic Concordance and Patient Satisfaction in Otolaryngology

**DOI:** 10.1002/oto2.70311

**Published:** 2026-11-06

**Authors:** Abigail G. McIntosh, Artemis Markopoulos, Julio A. Roque Buenrostro, Katie Holland, William B. Cohen, Mihir K. Bhayani, Vanessa C. Stubbs

**Affiliations:** ^1^ Department of Otorhinolaryngology–Head and Neck Surgery Rush Medical College, Rush University Medical Center Chicago Illinois USA; ^2^ Department of Otorhinolaryngology–Head and Neck Surgery Medical College of Wisconsin Milwaukee Wisconsin USA; ^3^ Department of Otorhinolaryngology–Head and Neck Surgery Rush University Medical Center Chicago Illinois USA

**Keywords:** diversity, otolaryngology, patient satisfaction, Press Ganey

## Abstract

**Objectives:**

Determine whether demographic concordance in age, gender, and race within physician‐patient dyads, as well as physician gender, significantly impacts patient satisfaction within otolaryngology.

**Study Design:**

Retrospective survey study from June 1 to December 31, 2023.

**Setting:**

Tertiary otolaryngology department at an academic hospital.

**Methods:**

Press Ganey survey responses from new otolaryngology patients were reviewed. Physician demographics were collected via survey. Electronic medical record review identified patient's providing physician and patient demographics: race, age, and gender. Then, physician‐patient demographic concordance or discordance was documented. Statistical analysis was performed using Stata statistical software (version 18; StataCorp.) with an alpha value of 0.05. Student's *t*‐test was used to compare quantitative variables.

**Results:**

378 otolaryngology patients were included. Patients reported higher satisfaction in gender concordant physician‐patient dyads across 4 of 7 analyzed Press Ganey questions (*P* < .05). Analysis of race and age concordance in physician‐patient dyads yielded no significant difference in satisfaction (*P* > .05). No significant difference was found between the Press Ganey scores of female versus male physicians (*P* > .05). Overall, the study population lacked diversity when compared to the institution's patient population.

**Conclusion:**

Our study demonstrates significantly higher patient satisfaction amongst gender concordant physician‐patient dyads. However, our study population was relatively homogeneous, highlighting the importance of replicating this research across more diverse provider groups and broadening the range of patients who complete satisfaction surveys. These findings ultimately reinforce the case for greater gender equity in otolaryngology, alongside targeted training to help providers deliver high‐quality care to patients of all backgrounds.

The 2010 Affordable Care Act (ACA) propelled the American healthcare system away from fee‐for‐service reimbursement and towards a value‐based care model.^1^ While fee‐for‐service remains the prominent reimbursement system across the country, financial incentives of the ACA, including the Hospital Value‐Based Purchasing Program, shifted the focus of healthcare from volume to quality.^1^, ^2^, ^3^ This shift made patient satisfaction questionnaires, such as Press Ganey surveys, invaluable, providing hospitals with profitable insight into patient satisfaction.^4^

The Press Ganey survey was created in 1985 as a strategy to quantify patient satisfaction after a healthcare encounter. Today, over 50% of American hospitals utilize the survey to garner data‐driven insight into patient perception.^5^ Crucial to utilizing data collected from Press Ganey surveys is uncovering the factors most influential to patient satisfaction with providers. Previous literature has shown that race discordant physician‐patient pairs, or dyads, those in which patient and physician race are not aligned, are associated with lower patient satisfaction scores.^6^, ^7^ Notably, one study published in 2023 demonstrated that the likelihood of a physician receiving a top satisfaction score diminished by 11% in race‐discordant dyads.^7^ While this statistic is striking, it is important to note that over 70% of the surveyed patients in this study self‐identified as White.^7^ Conversely, the influence of gender discordance has revealed mixed results. In a 2021 study, female surgeons had a 17% lower chance of receiving top satisfaction scores to the survey question, “likelihood of recommending the surgeon,” than their male counterparts.^8^ On the contrary, a paper by Takesheta et al, revealed that gender concordance had no significant effect on patient satisfaction across all specialties within a hospital system.^6^ Similarly, a 2020 study found no difference in Press Ganey scores between male and female providers within otolaryngology.^9^

While research into patient satisfaction with respect to demographic concordance exists, the literature is limited, the patient populations investigated lacked diversity, and research into the impact of demographic concordance within otolaryngology has yet to be investigated. We aim to address this gap in the literature by investigating the impact of both patient and physician demographics on patient satisfaction within an otolaryngology department at an urban academic health system.

## Methods

### Study Design and Settings

This study was approved by the Rush University Institutional Review Board. The primary outcome of this retrospective review was to investigate the association between patient satisfaction, as denoted by Press Ganey surveys, and social demographic concordance amongst physician‐patient dyads at an otolaryngology department within a large academic health system. The secondary outcome was to explore whether specific physician genders were associated with higher patient satisfaction scores.

### Study Population and Questionnaire

All new patients, at least 18 years of age, receiving otolaryngology care between 6/1/2023 and 12/31/2023 across the Rush academic health system were eligible for this study. Exclusion criteria included established otolaryngology patients, patients under 18 years of age, patients who saw a care provider other than an attending physician, and patients whose corresponding physician did not complete a demographic survey. All attending adult otolaryngology physicians, across the health system, were eligible for inclusion in the study. Attending pediatric otolaryngologists were excluded given the age of their patient population.

Press Ganey surveys assess patient satisfaction after an appointment with a healthcare provider. Patients are asked to rate various survey items on a 5‐point Likert scale, with 1 indicating “very poor” and 5 indicating “very good.” Press Ganey then translates these responses into a standardized 0‐to‐100‐point scale. A higher total survey score indicates a higher level of patient satisfaction. This study examined the following Press Ganey survey items: care provider overall, explanations the care provider gave you about your problem or condition, concern the care provider showed for your questions or worries, care provider's efforts to include you in decisions about your care, likelihood of you recommending this care provider to others, care provider's discussion of any proposed treatment (options, risks, benefits, etc.), and general likelihood of recommending the practice to others. Across this academic medical center, Press Ganey surveys are sent to patients after their appointment through text, if the patient's mobile number is on file, or through email. If neither the mobile number nor email is on file, the patient does not receive a survey.

Individual Press Ganey surveys were linked to specific patients via the contact serial number (CSN) on completed surveys. Patient demographic data, including age, as well as self‐reported race and gender, were collected through electronic medical record (EMR) review. Additionally, EMR review was utilized to establish which attending physician provided care to each new patient.

Demographic data from otolaryngology attending physicians across the health system was obtained prospectively using a voluntary 5‐question demographic survey distributed through REDCap (Research Electronic Data Capture). This survey collected the following information: name, age, race, and gender. Physician recruitment was conducted through department‐wide emails. Two emails were sent over a 2‐month period before recruitment initially closed. Following a third reminder email, the survey was re‐opened for another 2 weeks and subsequently closed.

All study data were collected and managed using REDCap electronic data capture tools hosted at the Rush University Medical Center.^10^, ^11^ REDCap is a secure, web‐based application designed to support data capture for research studies, providing: (1) an intuitive interface for validated data entry; (2) audit trails for tracking data manipulation and export procedures; (3) automated export procedures for seamless data downloads to common statistical packages; and (4) procedures for importing data from external sources.

### Data Analysis

Statistical analysis was performed using the Stata statistical software (version 18; StataCorp.) with an alpha value of 0.05. Student's *t*‐test was used to compare quantitative variables including Press Ganey scores respective to physician‐patient dyads.

## Results

### Study Population

There were 378 otolaryngology patients included in the final analysis. Of the patient population studied, 68.3% identified as White (n = 258), 15.6% (n = 59) as Black/African American, and 2.9% (n = 11) as Asian. The majority of patients were between the ages of 60 to 69 (29.6%, n = 112) and 70 to 79 (30.4%, n = 115). Sixty percent of the cohort was female (n = 227). Table 1 shows all patient demographic data.

**Table 1:** Demographic Composition of Patients

Demographic	Patients (%) (n = 378)
Race	
American Indian/Alaskan Native	1 (0.3)
Asian	11 (2.9)
Black or African American	59 (15.6)
Native Hawaiian/Other Pacific Islander	1 (0.3)
White	258 (68.3)
Other/Unknown	53 (14)
Age	
18‐29	13 (3.4)
30‐39	22 (5.8)
40‐49	22 (5.8)
50‐59	55 (14.6)
60‐69	112 (29.6)
70‐79	115 (30.4)
80‐89	31 (8.2)
99‐100	8 (2.1)
Gender	
Female	227 (60.1)
Male	148 (39.2)
Nonbinary	3 (0.79)

Fifteen adult otolaryngology attending physicians across the department completed the physician demographic survey. The 2 pediatric otolaryngology attendings were excluded from the analysis. Of these physicians, 66.7% (n = 10) identified as White, and 26.7% (n = 4) as Asian. Regarding age, 33.3% were between 30 and 39 (n = 5) years old and another 33.3% were between 40 and 49 (n = 5). Of the remaining physicians, 26.7% were between 50 and 59 (n = 4), and 6.7% were between 60 and 69 (n = 1). The majority, 76.7% (n = 13), identified as male. Table 2 shows all physician demographic data.

**Table 2:** Demographic Composition of Physicians

Demographic	Physicians (%) (n = 15)
Race^a^	
Asian	4 (26.7)
White	10 (66.7)
Other	1 (6.7)
Age	
30‐39	5 (33.3)
40‐49	5 (33.3)
50‐59	4 (26.7)
60‐69	1 (6.7)
Gender	
Female	2 (13.3)
Male	13 (86.7)

^a^ No physicians self‐identified as Black or African American, American Indian or Alaska Native, Native Hawaiian or Other Pacific Islander.

### Press Ganey Results—Primary Outcome

Among gender concordant physician‐patient dyads, patients reported significantly higher satisfaction across 4 of the 7 analyzed Press Ganey questions compared to gender discordant dyads. With respect to gender, in response to the survey question, “care provider overall,” patients in concordant dyads reported a mean score of 97.77 [95% CI 96.62‐98.92] compared to 94.98 in discordant dyads [95% CI 93.06‐96.89] (*P* = .03). To the survey question, “explanations the care provider gave you about your problem or condition,” patients in concordant dyads reported a mean score of 97.99 [95% CI 96.79‐99.19] compared to 95.37 in discordant dyads [95% CI 93.55‐97] (*P* = .04). To the question, “concern the care provider showed for your questions or worries,” patients in concordant dyads reported a mean score of 97.99 [95% CI 96.79‐99.19] compared to 95.15 in discordant dyads [95% CI 93.34‐96.97] (*P* = .02). Finally, to the question, “general likelihood of recommending the practice to others,” patients in concordant dyads reported a mean score of 98 [95% CI 96.72‐99.28] compared to 94.69 in discordant dyads [95% CI 92.86‐96.51] (*P* = .01) (Table 3).

**Table 3:** Gender Concordant and Discordant Press Ganey Scores

Press Ganey Scores						
Press Ganey Item	Concordant Group		Discordant Group		Mean difference (SE)	*P*‐value
	Mean (SD)	95% CI	Mean (SD)	95% CI		
Physician overall	97.77 (7.1)	(96.62‐98.92)	94.98 (14.65)	(93.06‐96.89)	2.79 (1.29)	.03
Explanations physicians provide to patients	97.99 (7.42)	(96.79‐99.19)	95.37 (13.94)	(93.55‐97.2)	2.61 (1.24)	.04
Concern physicians showed for patient's questions	97.99 (7.42)	(96.79‐99.19)	95.15 (13.86)	(93.34‐96.97)	2.83 (1.24)	.02
Physician's efforts to include patients in decisions	97.47 (8.62)	(96.07‐98.87)	94.98 (14.2)	(93.11‐96.85)	2.49 (1.3)	.06
Likelihood of your recommending physician to others	97.97 (7.99)	(96.67‐99.27)	95.67 (14.39)	(93.78‐97.56)	2.31 (1.3)	.08
Physician's discussion of treatments	97.09 (9.52)	(95.53‐98.65)	95.05 (14.78)	(93.09‐97)	2.04 (1.38)	.14
General likelihood of recommending	98 (7.94)	(96.72‐99.28)	94.69 (13.92)	(92.86‐96.52)	3.31 (1.25)	.01

Abbreviation: CI, confidence interval.

Across the remaining Press Ganey survey questions, there were no significant differences in patient satisfaction between race, gender, or age concordant versus discordant patient‐physician dyads (3, 4, 5).

**Table 4:** Age Concordant and Discordant Press Ganey Scores

Press Ganey Scores						
Press Ganey Item	Concordant Group		Discordant Group		Mean difference (SE)	*P*‐value
	Mean (SD)	95% CI	Mean (SD)	95% CI		
Physician overall	94.32 (13.7)	(89.76‐98.89)	96.28 (12.14)	(94.98‐97.57)	−1.95 (2.13)	.36
Explanations physicians provide to patients	94.59 (13.35)	(90.14‐99.05)	96.61 (11.68)	(95.36‐97.86)	−2.01 (2.05)	.33
Concern physicians showed for patient's questions	93.92 (13.7)	(89.35‐98.49)	96.53 (11.58)	(95.3‐97.77)	−2.62 (2.04)	.20
Physician's efforts to include patients in decisions	94.59 (13.35)	(90.14‐99.05)	96.12 (12.23)	(94.81‐97.43)	−1.52 (2.14)	.48
Likelihood of your recommending physician to others	94.59 (16.81)	(88.99‐100.2)	96.8 (11.71)	(95.54‐98.06)	−2.21 (2.13)	.30
Physician's discussion of treatments	92.57 (16.53)	(87.06‐98.08)	96.22 (12.5)	(94.87‐97.57)	−3.66 (2.24)	.10
General likelihood of recommending	95.95 (12.52)	(91.77‐100.12)	96.02 (11.96)	(94.74‐97.3)	−0.07 (2.08)	.97

Abbreviation: CI, confidence interval.

**Table 5:** Race Concordant and Discordant Press Ganey Scores

Press Ganey Scores						
Press Ganey Item	Concordant Group		Discordant Group		Mean difference (SE)	*P*‐value
	Mean (SD)	95% CI	Mean (SD)	95% CI		
Physician overall	96.45 (12.17)	(94.7‐98.2)	95.72 (12.44)	(93.93‐97.51)	0.73 (1.27)	.56
Explanations physicians provide to patients	96.68 (11.22)	(95.06‐98.29)	96.14 (12.47)	(94.35‐97.94)	0.53 (1.22)	.66
Concern physicians showed for patient's questions	96.94 (11)	(95.36‐98.52)	95.61 (12.56)	(93.8‐97.42)	1.33 (1.22)	.28
Physician's efforts to include patients in decisions	96.47 (12.02)	(94.72‐98.22)	95.48 (12.65)	(93.66‐97.3)	0.99 (1.28)	.44
Likelihood of your recommending physician to others	96.79 (12.29)	(95.02‐98.56)	96.37 (12.34)	(94.59‐98.16)	0.42 (1.28)	.74
Physician's discussion of treatments	96.58 (11.95)	(94.84‐98.33)	95.14 (13.91)	(93.12‐97.15)	1.45 (1.35)	.28
General likelihood of recommending	95.77 (12.17)	(94.02‐97.51)	96.26 (11.85)	(94.55‐97.97)	−0.49 (1.24)	.69

Abbreviation: CI, confidence interval.

### Press Ganey Results—Secondary Outcome

Additionally, without regard for patient gender, to the prompt, “likelihood of recommending this care provider to others,” female physicians received a mean score of 97.7 [95% CI 94.14‐101.1], while male physicians received an average score of 96.5 [95% CI 95.15‐97.81] (*P* = .61).

## Discussion

This study investigated the influence of patient and physician demographic concordance on patient satisfaction, as indicated by Press Ganey surveys, among new otolaryngology patients at a diverse tertiary care center. Previous studies have examined the impact of demographic concordance on patient satisfaction, but prior results have been conflicting, and concordance studies in the field of otolaryngology have yet to be explored. Our findings reveal that gender concordance significantly correlates with increased patient satisfaction across the majority of analyzed Press Ganey items. This correlation was not seen across other demographic concordances, including race or age.

### Gender and Press Ganey Results

Our findings show a significant correlation between physician‐patient gender concordance and patient satisfaction across 4 of the 7 analyzed Press Ganey items. These results add to the relatively scarce, and conflicting literature on this topic. For example, Prasad et al reported no significant difference in patient satisfaction within gender concordant versus discordant dyads across a sample of over 12,000 primary care Medicaid patients in Connecticut.^12^ A cross‐specialty study, analyzing over 100,000 Press Ganey surveys, revealed similar results, finding no significant correlation between gender concordance and higher reported patient satisfaction.^6^ Interestingly, Chekijian et al found that female patients in the emergency room reported a significant preference for female physicians yet reported lower satisfaction with these physicians when compared to male providers.^13^ Another cross‐specialty study echoed these results, revealing that female patients preferred same gender physicians.^14^ Our results, and the prior literature mentioned, emphasize the preference, specifically among female patients, for same gender physicians. This preference highlights the importance of female representation across medical specialties. In otolaryngology, this idea is especially poignant, as most practicing otolaryngologists remain male.^15^ While female representation within the specialty has steadily increased, from 11.2% in 2008 to 19% in 2022, the majority of otolaryngologists currently in practice remain predominately male.^15^, ^16^

Additionally, most existing literature on the impact of physician gender on patient satisfaction fails to analyze the influence of physician‐patient concordance. Instead, most studies focus on the impact of physician gender, regardless of patient demographic. For example, 2 studies found that female obstetrics and gynecology physicians and female surgeons were less likely to receive top satisfaction scores when compared to their male colleagues.^8^, ^17^ On the contrary, our study revealed no significant difference between the average satisfaction score for female versus male physicians to the same prompt, “general likelihood of recommending the physician to others.” While no significant difference existed between genders, only 2 of the 15 attendings included in our study were female, and only 32 of the patient surveys analyzed were for female physicians (full gender demographic data of each participating physician's patients can be found in Supplemental Table S1, available online). Thus, this small population makes drawing firm conclusions regarding the correlation between reported patient satisfaction and female physicians difficult. Interestingly, our results mirror the findings of a study published in 2020 by Tracy et al, which investigated the impact of physician gender on Press Ganey surveys across otolaryngology practices.^9^ This prior work found no significant difference between the Press Ganey scores of male and female providers.

### Race and Press Ganey Results

Furthermore, our study found no significant correlation between race‐concordant physician‐patient dyads and patient satisfaction across all analyzed Press Ganey survey items. These results differ from prior literature, which overwhelmingly suggests a positive influence of race concordance on patient satisfaction. Takeshita et al observed that racial/ethnic concordance amongst patients and their physicians resulted in a statistically significant increase in patient satisfaction.^6^ In fact, within the concordant group, 5.5% more physicians received top satisfaction scores when compared to discordant dyads.^6^ This data aligns with earlier research, which demonstrated that race‐concordant physician‐patient dyads led to higher patient satisfaction scores.^18^, ^19^

### Age and Press Ganey Results

Finally, our results showed no significant association between age concordance and patient satisfaction. This adds to the existing conflicting literature on this topic. A study published by Choi et al analyzed the impact of demographic concordance among a multicultural group of dermatology patients in Singapore.^19^ The study found no correlation between age concordance and overall patient satisfaction. In contrast, a multi‐national study by Eggermont et al found that age concordance was associated with increased satisfaction across all analyzed patient‐reported experience measures, except communication.^20^

### Demographics of Study Population

The lack of significant association between patient satisfaction and demographic concordance in this study, outside of gender, may be partially attributable to the absence of diversity within the study population. This study was conducted across a large academic otolaryngology department. This hospital system cares for a diverse patient population, as demonstrated by the Rush 2018 Health Equity Report, which found that of patients seen at the institution's outpatient specialty clinics, 25% identified as non‐Hispanic Black, 15% as Hispanic, 53% as non‐Hispanic White, and 7% as other/unknown.^21^ Yet, the patient population included in this study was overwhelmingly White, at 68.3%, with only 15.6% of patients self‐identifying as Black/African American. This lack of racial representation prevents an objective analysis of the impact of demographic concordance on patient satisfaction and sheds light on the shortcomings of patient satisfaction surveys.

Prior literature has warned about the usefulness of Press Ganey results as an indicator for hospital reimbursement and physician compensation. For example, Weir et al examined Press Ganey survey responses from an orthopedic surgery department and found a response rate of only 13.5%.^22^ Similar to our findings, Weir also reported that those who did respond were predominantly White, at 72%, whereas non‐respondents were more likely to be Black.^22^ Additionally, DeLoughery et al analyzed patient satisfaction survey data across 2 large health systems and found that the survey population was again overwhelmingly White, at 77%.^23^ These prior studies, and our results, bring into question the utility of patient satisfaction surveys. Given the non‐response bias seen across studies, as well as the lack of diversity in respondents, using patient satisfaction surveys as a tool for hospital and physician compensation may be misleading. This warning is especially prudent when we consider the impact of physician demographics on satisfaction scores, especially when many institutions tie these scores to physician incentives. Notably, a study by Shaw et al found that while race‐concordant dyads led to higher patient satisfaction across all races, physicians of color received lower satisfaction scores even within concordant patient encounters.^7^

### Limitations

This study's limitations include its small physician sample size, lack of diverse patient respondents, and physician diversity, which make generalizability difficult. Specifically, the limited number of female physicians eligible for inclusion in this study influenced our results. Additionally, the study's exclusion of returning patients limits analyses of changing patient satisfaction over subsequent visits. Future longitudinal research investigating patient satisfaction across numerous appointments could investigate how patient perception changes over time. Furthermore, future research should further investigate which patient demographics complete satisfaction surveys and effective strategies to diversify respondents and decrease non‐respondent bias.

## Conclusion

Our study revealed a statistically significant association between gender concordance and increased patient satisfaction across the otolaryngology department of a large, urban, academic health system. This finding underscores the importance of equitable hiring practices, focused on increasing female representation, and provider education initiatives, centered on communication strategies amongst demographically discordant physician‐patient pairs. Additionally, our study population's demographic composition highlights the nonresponse bias of patient satisfaction surveys and the need to identify strategies to increase respondent diversity. Our results emphasize that even within a diverse urban medical center, satisfaction surveys overwhelmingly represent the opinion of White patients. Furthermore, our provider population lacked diversity, and future research should investigate the impact of more diverse provider populations on patient satisfaction survey results. As healthcare systems shift towards value‐based care models that attach financial incentives to patient satisfaction, we must re‐consider the validity of patient satisfaction surveys to ensure they accurately capture the opinions of all patients, regardless of demographics, and prioritize equitable hiring practices which increase diverse representation within otolaryngology.

## Author Contributions

**Abigail G. McIntosh,** MD, conceptualization, analysis, investigation, methodology, project administration, manuscript writing; **Artemis Markopoulos**, MD, conceptualization, analysis, investigation, methodology, project administration, manuscript writing; **Julio A. Roque Buenrostro,** MS, investigation, data curation, manuscript editing; **Katie Holland**, MD, supervision, manuscript writing, project administration; **William B. Cohen**, MD, analysis, software, data curation. **Mihir K. Bhayani**, MD, supervision, conceptualization, manuscript editing, resources. **Vanessa C. Stubbs**, MD, supervision, conceptualization, manuscript editing, resources.

## Disclosures

### Competing interests

None.

### Funding source

None.

## Supporting information


**Supplemental Table S1. Participating Physician's Demographics and the Gender Identity of Their Patients.** This table summarizes participating physician's demographic characteristics and the gender composition of their patient populations.
