# Supplementary material for: Representation Matters: Demographic Concordance and Patient Satisfaction in Otolaryngology
Source: OTO Open. 2026 Nov 6;10(4):e70311. doi: 10.1002/oto2.70311 (PMC13741144; doi:10.1002/oto2.70311)
Supplement: Supplementary file 1 [file OTO2-10-e70311-s001.docx]

Supplemental Table S1. Participating Physician’s Demographics and the Gender Identity of Their Patients

| Physician Demographics |  | | Patient Gender Demographics |  |  |
| --- | --- | --- | --- | --- | --- |
| Physician | Gender | Age Range (Years) | Male %  (n) | Female %  (n) | Other %  (n) |
|  |  |  |  |  |  |
| Physician 1 | Female | 60-69 | 48 (15) | 52 (16) | 0 (0) |
| Physician 2 | Male | 30-39 | 0 (0) | 100 (5) | 0 (0) |
|  |  |  |  |  |  |
| Physician 3 | Male | 30-39 | 50 (25) | 50 (25) | 0 (0) |
|  |  |  |  |  |  |
| Physician 4 | Male | 40-49 | 21(6) | 79 (22) | 0 (0) |
|  |  |  |  |  |  |
| Physician 5 | Male | 50-59 | 71 (12) | 29 (5) | 0 (0) |
|  |  |  |  |  |  |
| Physician 6 | Male | 30-39 | 41 (24) | 59 (34) | 0 (0) |
|  |  |  |  |  |  |
| Physician 7  Physician 8  Physician 9  Physician 10  Physician 11  Physician 12  Physician 13  Physician 14  Physician 15 | Male  Female  Male  Male  Male  Male  Male  Male  Male | 50-59  30-39  50-59  30-39  50-59  40-49  40-49  40-49  40-49 | 0 (0)  0 (0)  45 (30)  28 (9)  30 (9)  0 (0)  27 (8)  60 (3)  29 (7) | 0 (0)  100 (1)  55 (37)  72 (23)  67 (20)  0 (0)  67 (20)  40 (2)  71 (17) | 0 (0)  0 (0)  0 (0)  0 (0)  3 (1)  0 (0)  7 (2)  0 (0)  0 (0) |
